# 5,6,7,8-Tetra­hydro-[1,2,4]triazolo[5,1-*b*]quinazolin-9(4*H*)-one

**DOI:** 10.1107/S2414314623004091

**Published:** 2023-05-12

**Authors:** Walid Ettahiri, Amal Dalbouha, Abdesselam Baouid, Abdulsalam Alsubari, Joel T. Mague, Mustapha Taleb, Youssef Ramli

**Affiliations:** a Laboratory of Engineering, Electrochemistry, Modeling and Environment, Faculty of Sciences, Sidi Mohamed Ben Abdellah University, Fez, Morocco; b Laboratory of Molecular Chemistry, Faculty of Sciences Semlalia, Cadi Ayyad University, Marrakech, Morocco; c Laboratory of Medicinal Chemistry, Faculty of Clinical Pharmacy, 21 September University, Yemen; d Department of Chemistry, Tulane University, New Orleans, LA 70118, USA; e Laboratory of Medicinal Chemistry, Drug Sciences Research Center, Faculty of Medicine and Pharmacy, Mohammed V University in Rabat, Morocco; f Mohammed VI Center for Research and Innovation (CM6), Rabat 10000, Morocco; Sunway University, Malaysia

**Keywords:** crystal structure, π-stacking, hydrogen bonding, triazole, tetra­hydro­quinazoline

## Abstract

The triazole ring in the title mol­ecule, C_9_H_10_N_4_O, is not quite coplanar with the six-membered ring to which it is fused, the dihedral angle between the two least-squares planes being 2.52 (6)°. In the crystal, a layered structure is formed by N—H⋯N and C—H⋯O hydrogen bonds plus slipped π-stacking inter­actions, with the fused cyclo­hexene rings projecting to either side.

## Abstract 2

The triazole ring in the title mol­ecule is not quite coplanar with the six-membered ring to which it is fused, the dihedral angle between the two least-squares planes being 2.52 (6)°. In the crystal, a layer structure is formed by N—H⋯N and C—H⋯O hydrogen bonds plus slipped π-stacking inter­actions, with the fused cyclo­hexene rings projecting to either side.

## Graphical abstract

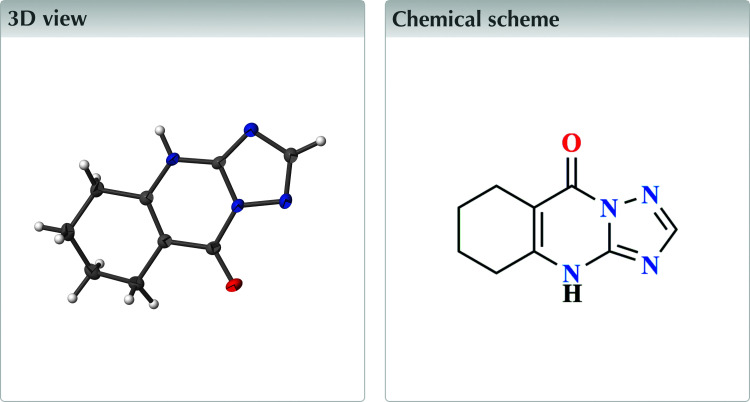

## Structure description

Compounds containing nitro­gen heterocycles make up a significant portion (approximately 60%) of small drug mol­ecules that have been approved by the FDA (Ramli & Essassi, 2015 ▸; Martins *et al.*, 2015 ▸). Quinazoline is a frequently occurring structural feature in natural products and pharmaceutically active mol­ecules., which possess a range of useful biological properties, including anti-SARS-CoV-2 (*e.g*. Karan *et al.*, 2021 ▸), anti­cancer (*e.g*. Zhao *et al.*, 2021 ▸), anti­viral (*e.g*. El-Shershaby *et al.*, 2021 ▸), anti­microbial, anti-inflammatory (*e.g*. Zhang *et al.*, 2020 ▸), and anti­fungal activities (*e.g*. Ibrahim *et al.*, 2021 ▸).

**Figure 1:**
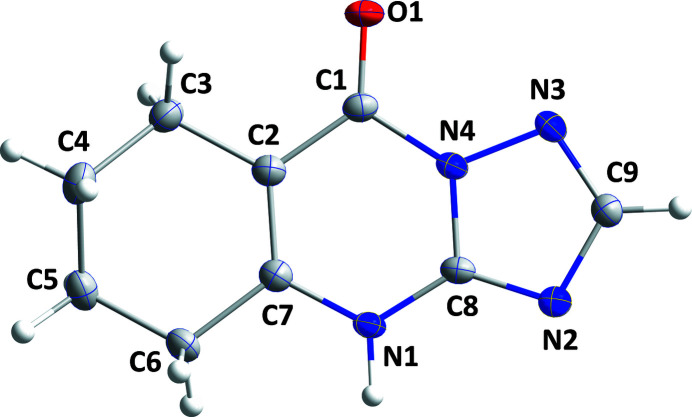
The title mol­ecule with labeling scheme and 50% probability ellipsoids.

**Figure 2:**
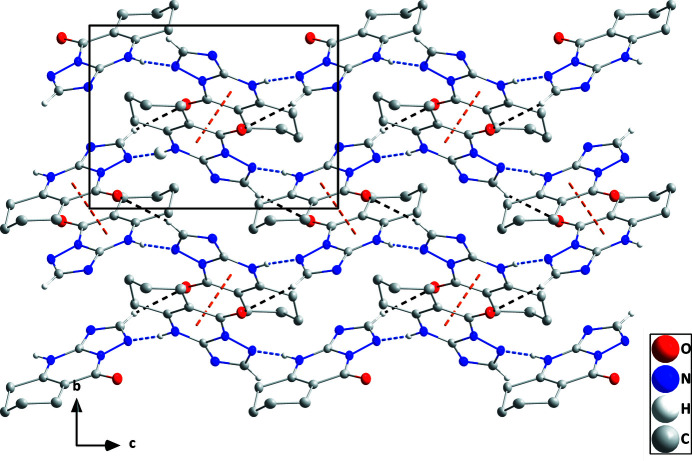
A portion of one layer viewed along the *a*-axis direction with N—H⋯N and C—H⋯O hydrogen bonds depicted by blue and black dashed lines, respectively. The slipped π-stacking inter­actions are depicted by orange dashed lines and non-inter­acting hydrogen atoms are omitted for clarity.

**Figure 3:**
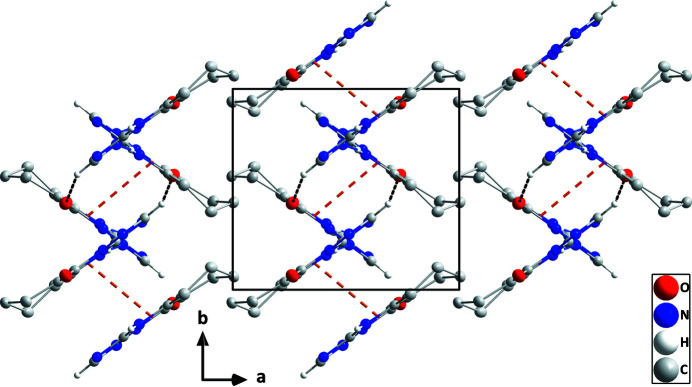
Packing viewed along the *c*-axis direction giving edge views of portions of three layers. Inter­molecular inter­actions are depicted as in Fig. 2 ▸ and non-inter­acting hydrogen atoms are omitted for clarity.

A puckering analysis of the C2–C7 ring of the title compound (Fig. 1 ▸) gave the parameters *Q* = 0.4922 (12) Å, θ = 129.71 (14)° and φ = 326.36 (18)°. This conformation is quite similar to a half-chair form. The C8/N2/C9/N3/N4 ring is closer to planarity than is the C1/C2/C7/N1/C8/N4 ring (r.m.s. deviations of the fitted atoms are 0.0128 and 0.0042 Å, respectively) and the dihedral angle between their mean planes is 2.52 (6)°. In the crystal, N1—H1⋯N3 hydrogen bonds (Table 1 ▸) form chains of mol­ecules extending along the *c*-axis direction, which are linked into layers parallel to the *bc* plane by weak C—H⋯O hydrogen bonds (Table 1 ▸ and Fig. 2 ▸). The layer formation is assisted by slipped π-stacking inter­actions between inversion-related C1/C2/C7/N1/C8/N4 rings [centroid–centroid distance = 3.4033 (6) Å, slippage = 0.96 Å]. The layers pack along the *a*-axis direction with van der Waals contacts between them (Fig. 3 ▸).

## Synthesis and crystallization

1*H*-1,2,4-Triazol-5-amine (0.5 g, 5.95 mmol) and ethyl 2-oxo-cyclo­hexa­necarboxyl­ate (0.951 ml, 5.95 mmol) were combined and heated under reflux in 10 ml of acetic acid for 1 h. The solid product obtained was recrystallized from ethanol solution to afford colorless crystals.

## Refinement

Crystal data, data collection and structure refinement details are summarized in Table 2 ▸.

## Supplementary Material

Crystal structure: contains datablock(s) global, I. DOI: 10.1107/S2414314623004091/tk4091sup1.cif

Structure factors: contains datablock(s) I. DOI: 10.1107/S2414314623004091/tk4091Isup2.hkl

Supporting information file. DOI: 10.1107/S2414314623004091/tk4091Isup3.cml

CCDC reference: 2259950

Additional supporting information:  crystallographic information; 3D view; checkCIF report

## Figures and Tables

**Table 1:** Hydrogen-bond geometry (Å, °)

*D*—H⋯*A*	*D*—H	H⋯*A*	*D*⋯*A*	*D*—H⋯*A*
N1—H1⋯N3^i^	0.919 (15)	1.907 (15)	2.8208 (12)	173.1 (13)
C9—H9⋯O1^ii^	0.95	2.57	3.3282 (12)	137

Symmetry codes: (i) 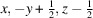 ; (ii) 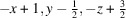 .

**Table 2:** Experimental details

Crystal data	
Chemical formula	C_9_H_10_N_4_O
*M*_r_	190.21
Crystal system, space group	Monoclinic, *P*2_1_/*c*
Temperature (K)	150
*a*, *b*, *c* (Å)	9.7925 (3), 7.9648 (3), 11.8039 (4)
β (°)	113.553 (1)
*V* (Å^3^)	843.95 (5)
*Z*	4
Radiation type	Cu *K*α
μ (mm^−1^)	0.86
Crystal size (mm)	0.36 × 0.15 × 0.12

Data collection	
Diffractometer	Bruker D8 VENTURE PHOTON 3 CPAD
Absorption correction	Multi-scan (*SADABS*; Krause *et al.*, 2015 ▸)
*T*_min_, *T*_max_	0.84, 0.91
No. of measured, independent and observed [*I* > 2σ(*I*)] reflections	18079, 1657, 1626
*R*_int_	0.021
(sin θ/λ)_max_ (Å^−1^)	0.618

Refinement	
*R*[*F* ^2^ > 2σ(*F* ^2^)], *wR*(*F* ^2^), *S*	0.030, 0.080, 1.07
No. of reflections	1657
No. of parameters	131
H-atom treatment	H atoms treated by a mixture of independent and constrained refinement
Δρ_max_, Δρ_min_ (e Å^−3^)	0.24, −0.19

Computer programs: *APEX4* and *SAINT* (Bruker, 2021 ▸), *SHELXT* (Sheldrick, 2015*a* ▸), *SHELXL2018/1* (Sheldrick, 2015*b* ▸), *DIAMOND* (Brandenburg & Putz, 2012 ▸) and *SHELXTL* (Sheldrick, 2008 ▸).

## full crystallographic data

### Crystal data

**Table:**

C_9_H_10_N_4_O	*F*(000) = 400
*M**_r_* = 190.21	*D*_x_ = 1.497 Mg m^−^^3^
Monoclinic, *P*2_1_/*c*	Cu *K*α radiation, λ = 1.54178 Å
*a* = 9.7925 (3) Å	Cell parameters from 9958 reflections
*b* = 7.9648 (3) Å	θ = 4.1–72.3°
*c* = 11.8039 (4) Å	µ = 0.86 mm^−^^1^
β = 113.553 (1)°	*T* = 150 K
*V* = 843.95 (5) Å^3^	Column, colourless
*Z* = 4	0.36 × 0.15 × 0.12 mm

### Data collection

**Table:**

Bruker D8 VENTURE PHOTON 3 CPAD diffractometer	1657 independent reflections
Radiation source: INCOATEC IµS micro—-focus source	1626 reflections with *I* > 2σ(*I*)
Mirror monochromator	*R*_int_ = 0.021
Detector resolution: 7.3910 pixels mm^-1^	θ_max_ = 72.4°, θ_min_ = 7.4°
φ and ω scans	*h* = −12→12
Absorption correction: multi-scan (*SADABS*; Krause *et al.*, 2015)	*k* = −9→9
*T*_min_ = 0.84, *T*_max_ = 0.91	*l* = −14→14
18079 measured reflections	

### Refinement

**Table:**

Refinement on *F*^2^	Primary atom site location: dual
Least-squares matrix: full	Secondary atom site location: difference Fourier map
*R*[*F*^2^ > 2σ(*F*^2^)] = 0.030	Hydrogen site location: mixed
*wR*(*F*^2^) = 0.080	H atoms treated by a mixture of independent and constrained refinement
*S* = 1.07	*w* = 1/[σ^2^(*F*_o_^2^) + (0.0413*P*)^2^ + 0.2836*P*] where *P* = (*F*_o_^2^ + 2*F*_c_^2^)/3
1657 reflections	(Δ/σ)_max_ = 0.001
131 parameters	Δρ_max_ = 0.24 e Å^−^^3^
0 restraints	Δρ_min_ = −0.19 e Å^−^^3^

### Special details

**Table:**

Experimental. The diffraction data were obtained from 18 sets of frames, each of width 0.5° in ω or φ, collected with scan parameters determined by the "strategy" routine in *APEX4*. The scan time was θ-dependent and ranged from 4 to 15 sec/frame.
Geometry. All esds (except the esd in the dihedral angle between two l.s. planes) are estimated using the full covariance matrix. The cell esds are taken into account individually in the estimation of esds in distances, angles and torsion angles; correlations between esds in cell parameters are only used when they are defined by crystal symmetry. An approximate (isotropic) treatment of cell esds is used for estimating esds involving l.s. planes.
Refinement. Refinement of F^2^ against ALL reflections. The weighted R-factor wR and goodness of fit S are based on F^2^, conventional R-factors R are based on F, with F set to zero for negative F^2^. The threshold expression of F^2^ > 2sigma(F^2^) is used only for calculating R-factors(gt) etc. and is not relevant to the choice of reflections for refinement. R-factors based on F^2^ are statistically about twice as large as those based on F, and R- factors based on ALL data will be even larger. H-atoms attached to carbon were placed in calculated positions (C—H = 0.95 - 0.98 Å) and included as riding contributions with isotropic displacement parameters 1.2 - 1.5 times those of the attached atoms. That attached to nitrogen was placed in a location derived from a difference map and was refined independently.

### Fractional atomic coordinates and isotropic or equivalent isotropic displacement parameters (Å^2^)

**Table:**

	*x*	*y*	*z*	*U*_iso_*/*U*_eq_	
O1	0.26314 (8)	0.43095 (10)	0.61185 (6)	0.0244 (2)	
N1	0.41264 (9)	0.32811 (10)	0.34454 (7)	0.0175 (2)	
H1	0.4489 (16)	0.3032 (18)	0.2857 (14)	0.034 (4)*	
N2	0.59918 (9)	0.16425 (10)	0.50516 (7)	0.0183 (2)	
N3	0.51360 (9)	0.22338 (11)	0.65438 (7)	0.0189 (2)	
N4	0.42719 (9)	0.30291 (10)	0.54577 (7)	0.0168 (2)	
C1	0.30105 (11)	0.40365 (12)	0.52669 (9)	0.0180 (2)	
C2	0.22902 (11)	0.46080 (12)	0.40036 (9)	0.0180 (2)	
C3	0.08681 (11)	0.55964 (13)	0.36710 (10)	0.0227 (2)	
H3A	0.111393	0.675596	0.398974	0.027*	
H3B	0.025843	0.507898	0.407433	0.027*	
C4	−0.00393 (12)	0.56563 (14)	0.22750 (10)	0.0245 (3)	
H4A	−0.050592	0.454782	0.198573	0.029*	
H4B	−0.084433	0.649834	0.208425	0.029*	
C5	0.09473 (12)	0.61123 (13)	0.15965 (9)	0.0236 (2)	
H5A	0.142182	0.721576	0.189077	0.028*	
H5B	0.032941	0.620358	0.069973	0.028*	
C6	0.21483 (11)	0.47830 (13)	0.18183 (9)	0.0215 (2)	
H6A	0.169975	0.379239	0.129506	0.026*	
H6B	0.292459	0.523786	0.156502	0.026*	
C7	0.28651 (11)	0.42377 (12)	0.31498 (9)	0.0172 (2)	
C8	0.48158 (10)	0.26443 (12)	0.45921 (8)	0.0160 (2)	
C9	0.61124 (11)	0.14311 (12)	0.62317 (9)	0.0185 (2)	
H9	0.686309	0.073934	0.680293	0.022*	

### Atomic displacement parameters (Å^2^)

**Table:**

	*U*^11^	*U*^22^	*U*^33^	*U*^12^	*U*^13^	*U*^23^
O1	0.0287 (4)	0.0296 (4)	0.0208 (4)	0.0032 (3)	0.0161 (3)	−0.0006 (3)
N1	0.0201 (4)	0.0205 (4)	0.0139 (4)	0.0001 (3)	0.0089 (3)	−0.0007 (3)
N2	0.0187 (4)	0.0190 (4)	0.0178 (4)	−0.0010 (3)	0.0079 (3)	−0.0009 (3)
N3	0.0206 (4)	0.0212 (4)	0.0143 (4)	−0.0004 (3)	0.0063 (3)	0.0019 (3)
N4	0.0188 (4)	0.0193 (4)	0.0133 (4)	−0.0007 (3)	0.0076 (3)	0.0002 (3)
C1	0.0195 (5)	0.0171 (5)	0.0196 (5)	−0.0020 (4)	0.0102 (4)	−0.0016 (4)
C2	0.0190 (5)	0.0170 (5)	0.0186 (5)	−0.0019 (4)	0.0083 (4)	−0.0008 (4)
C3	0.0218 (5)	0.0230 (5)	0.0254 (5)	0.0030 (4)	0.0115 (4)	0.0009 (4)
C4	0.0194 (5)	0.0222 (5)	0.0283 (6)	0.0025 (4)	0.0057 (4)	−0.0001 (4)
C5	0.0258 (5)	0.0211 (5)	0.0189 (5)	0.0010 (4)	0.0037 (4)	0.0013 (4)
C6	0.0239 (5)	0.0240 (5)	0.0157 (5)	0.0000 (4)	0.0069 (4)	0.0004 (4)
C7	0.0178 (5)	0.0161 (5)	0.0175 (5)	−0.0030 (3)	0.0067 (4)	−0.0011 (4)
C8	0.0182 (4)	0.0164 (5)	0.0152 (4)	−0.0036 (3)	0.0087 (4)	−0.0025 (3)
C9	0.0182 (5)	0.0188 (5)	0.0176 (5)	−0.0015 (4)	0.0063 (4)	0.0006 (4)

### Geometric parameters (Å, º)

**Table:**

O1—C1	1.2222 (12)	C3—C4	1.5287 (14)
N1—C8	1.3471 (12)	C3—H3A	0.9900
N1—C7	1.3724 (13)	C3—H3B	0.9900
N1—H1	0.919 (15)	C4—C5	1.5247 (15)
N2—C8	1.3258 (13)	C4—H4A	0.9900
N2—C9	1.3605 (12)	C4—H4B	0.9900
N3—C9	1.3192 (13)	C5—C6	1.5253 (14)
N3—N4	1.3762 (11)	C5—H5A	0.9900
N4—C8	1.3625 (12)	C5—H5B	0.9900
N4—C1	1.4135 (13)	C6—C7	1.5066 (13)
C1—C2	1.4449 (14)	C6—H6A	0.9900
C2—C7	1.3687 (14)	C6—H6B	0.9900
C2—C3	1.5087 (13)	C9—H9	0.9500
			
C8—N1—C7	120.21 (8)	C3—C4—H4B	109.5
C8—N1—H1	119.2 (9)	H4A—C4—H4B	108.1
C7—N1—H1	120.5 (9)	C4—C5—C6	110.58 (8)
C8—N2—C9	101.49 (8)	C4—C5—H5A	109.5
C9—N3—N4	101.96 (7)	C6—C5—H5A	109.5
C8—N4—N3	108.42 (8)	C4—C5—H5B	109.5
C8—N4—C1	125.93 (8)	C6—C5—H5B	109.5
N3—N4—C1	125.63 (8)	H5A—C5—H5B	108.1
O1—C1—N4	120.21 (9)	C7—C6—C5	112.64 (8)
O1—C1—C2	127.54 (9)	C7—C6—H6A	109.1
N4—C1—C2	112.23 (8)	C5—C6—H6A	109.1
C7—C2—C1	121.15 (9)	C7—C6—H6B	109.1
C7—C2—C3	121.96 (9)	C5—C6—H6B	109.1
C1—C2—C3	116.88 (9)	H6A—C6—H6B	107.8
C2—C3—C4	112.04 (8)	C2—C7—N1	121.71 (9)
C2—C3—H3A	109.2	C2—C7—C6	123.29 (9)
C4—C3—H3A	109.2	N1—C7—C6	114.96 (8)
C2—C3—H3B	109.2	N2—C8—N1	129.96 (9)
C4—C3—H3B	109.2	N2—C8—N4	111.37 (8)
H3A—C3—H3B	107.9	N1—C8—N4	118.66 (9)
C5—C4—C3	110.87 (8)	N3—C9—N2	116.74 (9)
C5—C4—H4A	109.5	N3—C9—H9	121.6
C3—C4—H4A	109.5	N2—C9—H9	121.6
C5—C4—H4B	109.5		
			
C9—N3—N4—C8	−0.54 (10)	C1—C2—C7—C6	179.19 (9)
C9—N3—N4—C1	177.90 (9)	C3—C2—C7—C6	0.39 (15)
C8—N4—C1—O1	−179.38 (9)	C8—N1—C7—C2	1.57 (14)
N3—N4—C1—O1	2.46 (15)	C8—N1—C7—C6	−176.34 (8)
C8—N4—C1—C2	1.78 (14)	C5—C6—C7—C2	13.45 (14)
N3—N4—C1—C2	−176.39 (8)	C5—C6—C7—N1	−168.67 (8)
O1—C1—C2—C7	178.33 (10)	C9—N2—C8—N1	−178.78 (10)
N4—C1—C2—C7	−2.93 (13)	C9—N2—C8—N4	0.72 (10)
O1—C1—C2—C3	−2.81 (15)	C7—N1—C8—N2	176.72 (9)
N4—C1—C2—C3	175.93 (8)	C7—N1—C8—N4	−2.74 (14)
C7—C2—C3—C4	16.83 (14)	N3—N4—C8—N2	−0.13 (11)
C1—C2—C3—C4	−162.02 (9)	C1—N4—C8—N2	−178.56 (8)
C2—C3—C4—C5	−47.48 (12)	N3—N4—C8—N1	179.43 (8)
C3—C4—C5—C6	62.20 (11)	C1—N4—C8—N1	1.00 (14)
C4—C5—C6—C7	−43.86 (11)	N4—N3—C9—N2	1.08 (11)
C1—C2—C7—N1	1.46 (15)	C8—N2—C9—N3	−1.16 (11)
C3—C2—C7—N1	−177.34 (9)		

### Hydrogen-bond geometry (Å, º)

**Table:**

*D*—H···*A*	*D*—H	H···*A*	*D*···*A*	*D*—H···*A*
N1—H1···N3^i^	0.919 (15)	1.907 (15)	2.8208 (12)	173.1 (13)
C9—H9···O1^ii^	0.95	2.57	3.3282 (12)	137

Symmetry codes: (i) *x*, −*y*+1/2, *z*−1/2; (ii) −*x*+1, *y*−1/2, −*z*+3/2.
